# Pruritus and brain tumours: A prospective and descriptive study

**DOI:** 10.1002/ski2.202

**Published:** 2022-12-22

**Authors:** Marie‐Anne Fardel, Emilie Brenaut, Dewi Guellec, Maxime Etienne, Maxime Fouchard, Romuald Seizeur, Laurent Misery

**Affiliations:** ^1^ Department of Dermatology University Hospital of Brest Brest France; ^2^ University of Brest LIEN Brest France; ^3^ Department of Neurosurgery University Hospital of Brest Brest France

## Abstract

**Background:**

Pruritus, especially localised to the nostrils, has been reported as a specific sign of brain tumours.

**Objectives:**

The main goal of this study was to estimate the prevalence of pruritus in a group of patients with brain tumours. The second outcome was to better characterise this pruritus with a specific questionnaire and a skin examination.

**Methods:**

From June 2020 to September 2021, all patients with a diagnosis of brain tumour were included in this prospective, monocentric study. If the patient suffered from pruritus, a dermatological examination was performed.

**Results:**

Two hundred patients with brain tumours were included. Thirty‐five of them suffered from pruritus (17.5%). Among them, 15 patients did not present with any skin disease, and 8 could have neuropathic pruritus according to the NP5 questionnaire. No patients presented with pruritus of the nostrils.

**Discussion:**

This study did not show clear evidence of specifically localised pruritus induced by brain tumours.

**Conclusion:**

Pruritus observed in patients with brain tumours seems not to be caused by the brain malignancies in most cases. The specific localization to the nostrils cannot be considered a specific marker.

## Graphical abstract

Pruritus, especially localised to the nostrils, has been reported as a specific sign of brain tumours by many authors. This prospective and descriptive study did not show clear evidence of specifically localised pruritus induced by brain tumours. Thus, pruritus of the nostrils should no longer be considered as a specific sign of brain tumours.

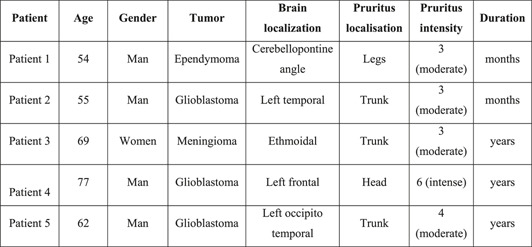

**What is already known about this topic?**

- Pruritus, especially of the nostrils, is considered as a specific sign of brain tumours.
- No rigourous study studied the prevalence and the characteristics of pruritus in a group of patient with brain tumour

**What does this study add?**

- Our study does not establish a clear association between brain tumours and pruritus.
- Pruritus specifically localised to the nostrils should no longer be considered a specific symptom of brain tumours.

## 1 INTRODUCTION

Pruritus is an unpleasant feeling that causes the desire to scratch. It is one of the most common symptoms in dermatology. Many skin diseases (such as eczema and psoriasis) and systemic disorders (such as cholestasis and renal failure) induce pruritis. Itch can also be caused by neuronal or glial damage, which constitutes neuropathic itch.^1^, ^2^ In this case, itch is often associated with other symptoms, such as pain, allodynia or paraesthesia, and its treatment is complex.^3^

Brain tumours have also been hypothesised to cause itch. Andreev and Petkov have reported that pruritus, especially pruritus of the nostrils, may be a sign of advanced brain tumours.^4^ In this study, out of 77 patients with brain tumours, 13 patients complained of pruritus; for seven of them, pruritus was specifically localised to the nostrils and was associated with tumours of the fourth ventriculi (a part of the brainstem).

Although pruritus of the nostrils is still described as a specific sign of brain tumours in several reference books in dermatology or oncology, the clinical association of brain tumours with itch remains rather poorly characterised with respect to its prevalence, the type of pruritus, that is, localised or diffuse, and the nature of the brain tumours. The present study was designed to gain insights into these points by analysing the presence and characteristics of pruritus in a prospective cohort of 200 adult patients exhibiting primary or secondary benign or malignant brain tumours.

## 2 PATIENTS AND METHODS

### 2.1 Patients

This prospective, monocentric and noninterventional study included all consecutive patients from the Department of Neurosurgery of the University Hospital of Brest presenting with brain tumours. The study was approved by the research ethics committee (Comité de protection des personnes Ile de France II 2018‐A01998‐47) and was registered on clinicaltrials. gov (NCT03673878).

The inclusion criteria were as follows: age 18 and older, malignant or benign brain tumour, at discovery or later, and primary or secondary brain tumours confirmed by anatomopathological sample and magnetic resonance imaging (MRI). Exclusion criteria were age <18 years, participation refusal, adults not able to answer questions, no cerebral MRI, and previous treatment on the brain (radiotherapy or surgery).

### 2.2 Data

The primary study endpoint was the presence of pruritus for at least 1 week. When a patient complained of pruritus, a dermatological examination was performed by a dermatologist to look for a skin disease. Other possible causes of pruritus, such as medications or biological disorders, were recorded.

The secondary outcome was to better characterise pruritus in patients with brain tumours. In patients with pruritus, a questionnaire was completed concerning the intensity of pruritus, localization, date of onset, NP5 questionnaire, medical or biological history and treatment. Intensity was estimated on a Visual Numeric Scale from 1 to 10 (VNS). NP5 consists of a list of five questions developed for the diagnosis of neuropathic pruritus. The presence of twinges, absence of burning, worsening with activity, non‐worsening with stress and relief with cold ambient temperature are factors associated with neuropathic pruritus. A score of two criteria out of five was optimal to discriminate neuropathic pruritus from nonneuropathic pruritus.^5^ The main aetiology of pruritus was noted.

### 2.3 Sample size

In the study by Andreev and I. Petkov, the prevalence of pruritus was 17%.^4^ According to our group's statistician, a total of 200 patients was estimated to be necessary to estimate pruritus prevalence with a precision of 5% (half the width of the confidence interval), based on an expected value of approximately 17%. Neurosurgeons determined that the period necessary to recruit 200 patients with brain tumours in the Department of Neurosurgery of the University Hospital of Brest was one year.

### 2.4 Statistical analysis

Descriptive statistics included the mean, median, standard deviation, minimum and maximum for continuous variables and percentages for qualitative variables. Pruritus prevalence for all brain tumours and for each subgroup was estimated with a 95% confidence interval, calculated with a binomial distribution. Qualitative variables were compared between groups using a chi‐square test. The criterion of significance was *p* value < 0.05.

## 3 RESULTS

### 3.1 Characteristics of the population

A total of 200 patients were included between June 2020 and September 2021 (Table 1). The median age was 62.5 years; the oldest patient was 85, and the youngest was 18. Men and women were equally represented (46.5% women and 53.5% men). Glioblastomas (33.5%), brain metastases (21%) and meningiomas (20.5%) were the most frequent brain tumours. A large number of patients had only one brain tumour localization (93.5%). Various localisations were reported, and the most represented were the right frontal (17.5%), cerebellar (13%), left frontal (11%) and pituitary (9%) regions (Table 1).

**TABLE 1:** Characteristics of patients with brain tumours

Characteristics	Patient number: *n* (%)
Number of patients included in the study	*n* = 200
Gender	Women: *n* = 93 (46.5)
	Men: *n* = 107 (53.5)
Age	<40: *n* = 11 (5,5)
	41–50: *n* = 32 (16)
	51–60: *n* = 32 (16)
	61–70: *n* = 57 (28.5)
	71–80: *n* = 59 (29.5)
	>80: *n* = 9 (4.5)
Number of tumours	Unique: *n* = 187 (93.5)
	Multiple: *n* = 13 (7.5)
Subtype of tumours	Glioblastoma: *n* = 67 (33.5)
	Metastasis: *n* = 42 (21)
	Meningioma: *n* = 41 (20.5)
	Adenoma: *n* = 18 (9)
	Lymphoma: *n* = 9 (4.5)
	Other: *n* = 7 (3.5)
	Oligodendroglioma: *n* = 6 (3)
	Astrocytoma: *n* = 5 (3)
	Ependymoma: *n* = 3 (1.5)
	Chordoma: *n* = 2 (1)
Localization	Right frontal: *n* = 35 (17.5)
	Other: *n* = 35 (17.5)
	Cerebellar: *n* = 26 (13)
	Left frontal: *n* = 22 (11)
	Pituitary: *n* = 18 (9)
	Left parietal: *n* = 8 (4)
	Left temporal: *n* = 8 (4)
	Right temporal: *n* = 7 (3.5)
	Corpus callosum: *n* = 6 (3)
	Right fronto parietal: *n* = 6 (3)
	Left occipital: *n* = 4 (2)
	Left fronto parietal: *n* = 4 (2)
	Right occipital: *n* = 3 (1.5)
	Ethmoidal: *n* = 3 (1.5)
	Cerebellopontine angle: *n* = 3 (1.5)

### 3.2 Patients with pruritus

Thirty‐five patients out of 200 complained of pruritus (17.5%). A major portion of them (82.8%) presented with pruritus for more than a year (Table 2). Localization of pruritus was mostly on the trunk (28.6%) and on the head (28.6%). No patient reported localised pruritus in the nostrils.

**TABLE 2:** Characteristics of pruritus

Characteristics		Total *N* = 35 (%)	Dermatologial pruritus *N* = 20 (%)	Pruritus *sinae materia N* = 15 (%)
Duration	>1 year	29 (82.8)	17 (85)	12 (80)
	>1 month	6 (17.2)	3 (15)	3 (20)
Localization	Head	10 (28.6)	8 (40)	2 (13.3)
	Trunk	10 (28.6)	4 (20)	6 (40)
	Legs	5 (14.3)	2 (10)	3 (20)
	Arms	4 (11.4)	2 (10)	2 (13.3)
	Extended	3 (8.6)	2 (10)	1 (6.7)
	Other	3 (8.6)	2 (10)	1 (6.7)
VNS	<3	7 (20)	3 (15)	4 (26.7)
	3–5	20 (57.1)	12 (60)	8 (53.3)
	5	5 (14.3)	3 (15)	2 (13.3)
	3	3 (8.6)	2 (10)	1 (6.7)
NP5	0–1	27 (77.1)	14 (70)	13 (86.7)
	≥2	8 (22.9)	6 (30)	2 (13.3)

The intensity was moderate (VNS 3–5) in more than half of the patients (57.1%). A total of 22.9% of the patients exhibiting pruritus met two criteria of the NP5, compatible with neuropathic pruritus.

After physical examination, a dermatological diagnosis explaining this pruritus was found in 57.1% of patients with pruritus. Seborrhoeic dermatitis (22.7%), itchy seborrhoeic keratoses (14.3%), and eczema (8.6%) were the most frequent aetiologies for pruritus (Table 3). Topography of the pruritus was thus related to the dermatitis; for example, it was localised at the head for patients with seborrhoeic dermatitis.

**TABLE 3:** Dermatological diagnoses causing pruritus

Diagnosis	*N* = 20
Seborrhoeic dermatitis	8
Seborrhoeic keratosis	5
Eczema	3
Prurigo	1
Zona	1
Cheiletis	1
Psoriasis	1

A total of 15 patients with pruritus had no dermatological diagnosis explaining this symptom. Other aetiologies of pruritus were investigated. Five patients were 70 years old or older and complained of pruritus associated with xerosis, suggesting senile pruritus.^6^ Medications associated with pruritus were found in one patient (angiotensin receptor blocker). Biological disorders such as renal failure were found in two patients, and cholestasis was reported in two others.

Finally, 5 patients had pruritus without any dermatological or systemic aetiology, and a link with brain tumour localization was therefore hypothesised. For 4 of them, the diagnosis of the brain tumour was recent (<1 month). The brain localization was different for each patient (Table 4). Additionally, the subtypes of brain tumours varied (3 glioblastomas, 1 ependymoma, 1 meningioma). Pruritus intensity was moderate for 4 of them, and its localization also varied (3 cases were located on the trunk, 1 case on the legs and 1 on the scalp).

**TABLE 4:** Characteristics of patients with pruritus without clear aetiology

Patient	Age	Gender	Tumour	Brain localization	Pruritus localisation	Pruritus intensity	Duration
Patient 1	54	Man	Ependymoma	Cerebellopontine angle	Legs	3 (moderate)	months
Patient 2	55	Man	Glioblastoma	Left temporal	Trunk	3 (moderate)	months
Patient 3	69	Women	Meningioma	Ethmoidal	Trunk	3 (moderate)	years
Patient 4	77	Man	Glioblastoma	Left frontal	Head	6 (intense)	years
Patient 5	62	Man	Glioblastoma	Left occipito temporal	Trunk	4 (moderate)	years

Concerning the 8 patients with neuropathic pruritus according to the NP5, 6 of them had a dermatological diagnosis explaining their symptoms. For two of them, pruritus was potentially related to their brain tumour.

## 4 DISCUSSION

To the best of our knowledge, this study is the first to evaluate the prevalence of pruritus and its characteristics in a large cohort of patients with brain tumours. The prevalence of pruritus in our study was 17.5%. A similar frequency of pruritus was previously reported in the general population.^7^ Other studies found a frequency of approximately 10% for acute itch in the general population.^8^, ^9^, ^10^ Because itch is often associated with psychological factors,^11^ negative life events such as the announcement of a brain tumour should favour itch and thus might explain the higher frequency of pruritus in our group of patients compared to the general population.

After a skin examination, a dermatological diagnosis could explain the majority of the cases of pruritus (57%). Pruritus is a common symptom but is often overlooked in clinical routine. However, many skin diseases induce itch, and a dermatological examination leads to the identification of an aetiology for pruritus in a large majority of patients.

In our study, pruritus was more frequent among elderly people: 80% of patients complaining of pruritus were 61 years old or older. Pruritus can be a consequence of physiological changes that occur with ageing^6^, ^7^, ^8^, ^9^, ^10^, ^11^, ^12^ and dysfunction of the cutaneous barrier.^13^ This result in our study is thus consistent with what is known in the scientific literature.

In our study, many patients with brain tumours described pruritus of the head, but none of them reported pruritus specifically localised to the nostrils. Pruritus of the head could be explained by the high prevalence of seborrhoeic dermatosis (22,9%). Seborrhoeic dermatosis has been reported to be associated with various neurological disorders as well as emotional stress.^14^, ^15^ Some studies have argued that autonomic disorders induced by brain disorders could explain the increased production of sebum. Other studies suggest that reduced movements induced by neurological disorders lead to a lack of hygiene that promotes Malassezia growth involved in seborrhoeic dermatosis.^16^ In our study, the relatively high prevalence of seborrhoeic dermatosis (22,9%) may thus be explained by neurodegenerative disorders and the emotional stress induced by the brain tumours.

Several authors have reported an association between pruritus and brain tumours. Gail Summers^17^ reported two cases of children with brainstem glioma with facial itch as a prominent clinical symptom. Andreev et al.^4^ described severe and persistent pruritus of the nostrils as a specific sign of advanced brain tumours localised to the brainstem. Another author reported a case of an intramedullary cavernous haemangioma inducing central neuropathic itch.^18^ Thus, pruritus is suspected to be specifically induced by brain tumours in those studies. In our study, only 5 patients out of 200 suffered from pruritus with no clear aetiology, and pruritus may thus be related to their brain tumour, while 8 presented with an NP5 score suggesting a neuropathic itch. Localization of these brain tumours and the subtype of tumour were variable, and no link could be established between brain localization and pruritus. Additionally, no pruritus specifically localised to the nostrils was reported in our study, unlike in the Andreev study.^4^ It is noteworthy that nostril pruritus can be secondary to dermatological causes such as seborrhoeic dermatitis or eczema, but also to allergic causes such as allergic rhinitis. Also, pruritus of the nostrils specifically for tumours of the fourth ventricle have been reported by Andreev et al^4^; however, in our study, only 2 patients had tumours of the fourth ventricle and none of them had pruritus without aetiology found. Therefore, no clear evidence was found between the occurrence of brain tumours and specifically localised pruritus in our prospective study of 200 patients.

The mechanisms of pruritus at both the peripheral and central levels have been studied.^19^, ^20^, ^21^ At the central level, many brain areas are involved in itch processing, including the sensory, emotional and motivational pathways. The sensory component involves various brain regions, such as the thalamus, the praecuneus and the somatosensory cortex.^22^ Therefore, these previous studies demonstrate that central mechanisms involved in pruritus are complex and not completely characterised. As pruritus results from multicentered brain areas, it is difficult to make a direct association between a unique brain tumour and induced pruritus.

Our study presents some limitations. Our prospective descriptive study offers interesting data, but a comparison of the prevalence of pruritus among patients with brain tumours and the general population in a case‒control study would be necessary. Also, it might be interesting to look retrospectively at a group of patients with pruritus of the nostrils for a possible association with the presence of a brain tumour. Furthermore, it cannot be excluded that the exact localization of the brain tumour is a key‐factor to trigger pruritus. To investigate this hypothesis, a large cohort of patients with brain tumours and pruritus without known aetiology would be needed.

In conclusion, our study does not establish a clear association between brain tumours and pruritus, although some rare cases of pruritus could be associated with these tumours. Therefore, pruritus specifically localised to the nostrils should no longer be considered a specific symptom of brain tumours.

## CONFLICT OF INTEREST

The authors have no relevant financial or non‐financial interests to disclose.

## AUTHOR CONTRIBUTIONS

**Marie‐Anne Fardel**: Formal analysis (Equal); Investigation (Equal); Resources (Equal); Software (Equal); Writing – original draft (Equal); Writing – review & editing (Equal). **Emilie Brenaut**: Conceptualization (Equal); Methodology (Equal); Supervision (Equal); Validation (Equal). **Dewi Guellec**: Conceptualization (Equal); Data curation (Equal); Project administration (Equal). **Maxime Etienne**: Conceptualization (Equal); Methodology (Equal); Project administration (Equal). **Maxime Fouchard**: Conceptualization (Equal); Investigation (Equal); Methodology (Equal); Project administration (Equal); Resources (Equal); Software (Equal). **Romuald Seizeur**: Conceptualization (Equal); Data curation (Equal); Project administration (Equal). **Laurent Misery**: Conceptualization (Equal); Project administration (Equal); Resources (Equal); Supervision (Equal); Validation (Equal); Writing – original draft (Equal); Writing – review & editing (Equal).

## ETHICS STATEMENT

The study was approved by the research ethics committee (Comité de Protection des Personnes Ile de France II 2018‐A01998‐47) and was registered on clinicaltrials. gov (NCT03673878). The study was performed in line with the principles of the Declaration of Helsinki.

## Data Availability

The dataset analysed during the current study are available from the corresponding author on reasonable request.
